# Optimal Ablation Techniques for Ventricular Tachycardia Management

**DOI:** 10.19102/icrm.2018.090101

**Published:** 2018-01-15

**Authors:** Jose M. Sanchez, Chen Yuan, Henry H. Hsia

**Affiliations:** ^1^Cardiac Electrophysiology Section, University of California, San Francisco, San Francisco, CA, USA; ^2^Department of Cardiology, Cangzhou Central Hospital, Cangzhou, Hebei Province, China

**Keywords:** Ablation, image integration, mapping, substrate characterization

## Abstract

Ventricular arrhythmias arise from complex electroanatomical substrates in patients with structural heart disease. There have been significant advancements in technologies and techniques for ventricular tachycardia catheter ablation. A systematic approach to mapping and ablation is paramount, and catheter ablation has shifted to be a potential first-line therapy for patients needing early intervention, particularly for those with post-infarction arrhythmias. Furthermore, imaging integration, coupled with a systematic, detailed substrate characterization, has shown promise and provides a safe and effective approach for long-term arrhythmia control.

## Introduction

Sustained ventricular tachycardia (VT) is a notable cause of morbidity and mortality in patients with structural heart disease, claiming up to 400,000 lives per year in the United States alone.^1^ Implantable cardioverter-defibrillators (ICDs) are effective in terminating VT, and in reducing the risk of arrhythmic death.^2,3^ However, ICD shocks can lead to a significant reduction in quality of life, and have been associated with increased morbidity and mortality.^4,5^ Although antiarrhythmic drugs (such as amiodarone) may be effective in preventing arrhythmia recurrences and ICD-delivered therapies, they include an increased risk of drug-related adverse effects and non-recurrences and ICD-delivered therapies, they include an cardiac mortality.^6^ With the continued development of ablation strategies and technologies, catheter ablation of VT is becoming an increasingly acceptable therapy in the management of VT, particularly in those patients with ischemic heart disease and prior infarctions.^7^

### Arrhythmogenic substrate

The field’s understanding of the mechanisms of VT has come primarily from the management of patients with ischemic heart disease and myocardial infarctions. The majority of VT cases in patients with structural heart disease are due to scar-based reentry. In those individuals with prior myocardial infarctions, VT commonly originates from the subendocardial region of the infarcted myocardium. These areas of myocardial scar alter the impulse propagation, thereby producing slow and anisotropic conduction.^8,9^ Such delayed local activation can be detected by signal-averaged electrocardiography, presenting as local abnormal ventricular activities (LAVAs) with fractionated, split, and late potentials recorded during electroanatomic mapping.^10,11^ Successful subendocardial resection can abolish local abnormal ventricular activities and late potentials, with elimination of post-infarction VT inducibility.^11^

**Figure 1:**
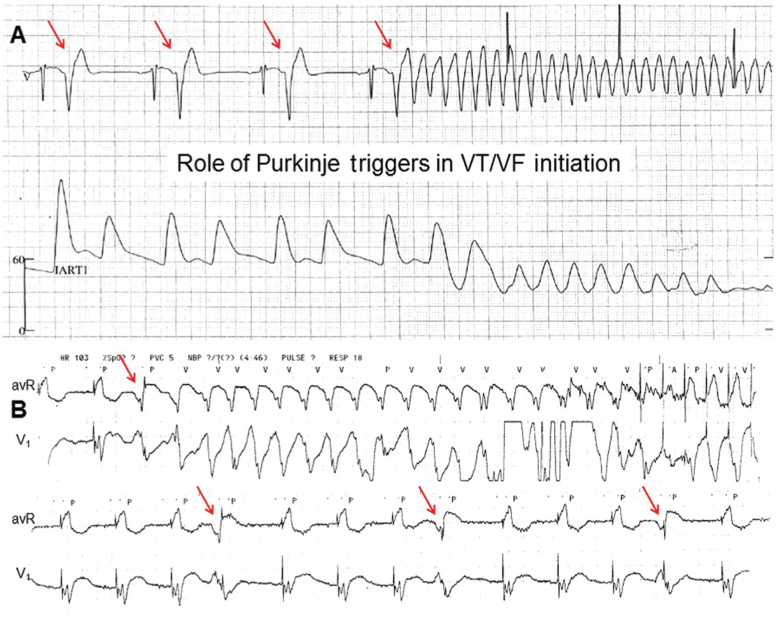
Purkinje-related ventricular tachyarrhythmias in ICM. **A:** Purkinje triggers manifested as closely coupled premature ventricular complexes (PVCs; arrows) for polymorphic VT or VF initiation. **B:** Purkinje fibers arborized along the infarct border may also serve as either a localized microreentrant or part of a macroreentrant circuit using the subendocardial scar substrate for monomorphic VT. This may manifest as frequent monomorphic PVCs (arrows). ICM: ischemic cardiomyopathy; VF: ventricuventricular fibrillation; VT: ventricular tachycardia.

Multiple morphologies of spontaneous or inducible VTs were observed in a majority (60%–80%) of post-infarction patients who underwent catheter ablation.^12,13^ The reentrant circuits associated with different VT morphologies are rarely from disparate scar locations and are commonly within the same infarcted region.^12,14^ Furthermore, a “shared” isthmus is present in approximately 43% of patients with multiple VTs.^15^ In addition to scar-based reentry, the subendocardial Purkinje fibers also play an important role in ventricular arrhythmia in these patients. Purkinje fibers arborize along the border zone of the subendocardial scar, and may serve as a localized reentry substrate for monomorphic VT^16^ and also as triggers for polymorphic ventricular arrhythmias **(Figure 1)**.^17^

**Figure 2:**
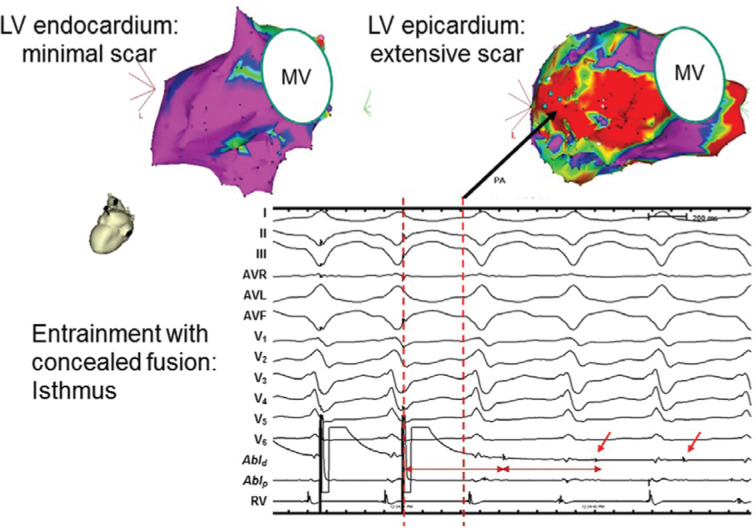
Endoepicardial mapping in patients with NICM. Endocardial and epicardial bipolar electrogram recordings were performed in a patient with NICM. The LV endocardial voltage showed minimal abnormal low-voltage areas, while the LV epicardial voltage showed extensive low-voltage scar located near the basal lateral LV. The endocardial and epicardial bipolar voltage color gradient is depicted. The purple areas represent normal tissue (endocardial amplitude ≥ 1.5 mV or epicardial amplitude > 1.0 mV) whereas dense scar is depicted in red (amplitude < 0.5 mV). The border zone is defined as areas with the color gradient being between red and purple. Activation mapping during sustained VT demonstrated an early, mid-diastolic potential located at the LV lateral epicardium (arrow). Overdrive pacing at this site showed entrainment with concealed fusion, consistent with an epicardial isthmus site. LV: left ventricular; MV: mitral valve; NICM: nonischemic cardiomyopathy; VT: ventricular tachycardia.

Nonischemic cardiomyopathy (NICM) constitutes a group of heterogeneous diseases resulting in myocardial dysfunction that is in the absence of significant coronary artery disease. In addition to Purkinje fascicular-related reentry and focal automaticity, the predominant mechanism (62%–89%) of VT in NICM remains as myocardial scar-based reentry.^18^ In contrast with patients with postinfarction ventricular arrhythmias, the majority of patients with NICM have only limited areas of endocardial scar. The scar location does not conform to the distribution of the coronary arteries, and is often located near the perivalvular region **(Figure 2)**.^19^ Delayed enhancement magnetic resonance imaging (MRI) and electroanatomic mapping has demonstrated the presence of mid-myocardial and epicardial low-voltage areas compared with the endocardial surface, located near the valve annulus.^15,20^

## Preprocedural planning

To maximize the success of VT ablation in patients with structural heart disease, a thorough preoperative assessment is essential. Careful attention should be paid to the 12-lead electrocardiograms (ECGs). The presence of Q-waves in sinus rhythm on the ECG provides a noninvasive clue for localization of myocardial scarring, and identifies a potential arrhythmia substrate in patients with ischemic cardiomyopathy (ICM). Careful analysis of the 12-lead ECG allows for localization of the “exit site” for ischemic VTs with a reasonable predictive accuracy (> 70%) to guide the initial mapping efforts.^21^ In the absence of sustained VT, the morphology of premature ventricular contractions can also be useful to locate the regions of potential arrhythmia substrate or to ascertain the possibility of Purkinje involvement. Furthermore, various QRS morphologic criteria have been used to distinguish endocardial from epicardial or non-endocardial sites of origins, especially for VTs associated with NICMs (see later discussion below).^22,24^

## Mapping strategies

Conventional mapping techniques, such as activation and/or entrainment mapping, identify the functional components and the critical isthmus of a reentrant VT circuit. Such mapping strategies, however, are dependent on the inducibility and hemodynamic tolerance of the arrhythmia. In clinical practice, the majority of induced VTs are poorly tolerated, and therefore activation and entrainment mapping have a limited role. A paradigm shift focusing more on a substrate-based mapping strategy is generally required for successful VT ablation.

The basis of electroanatomical characterization of the myocardial substrate relies on high-density endocardial bipolar voltage mapping during sinus or paced rhythm. Areas of the endocardium that are relatively healthy or “normal” have endocardial bipolar voltages greater than 1.5 mV, while areas with low voltage (< 0.5 mV) are arbitrarily classified as “dense scar.” Border zones are regions between healthy and scarred myocardium, and typically have bipolar voltages ranging from 0.5 mV to 1.5 mV.^25^ These predefined voltage criteria have been validated in animal models and human histopathology, and help to identify the location end and extent of endocardial scar.^26,27^

Based on detailed entrainment mapping of hemodynamically tolerated arrhythmias, most VT isthmus or entrance sites (84%) are identified within the dense scar (< 0.5 mV), and most of the exits are located in the border zone (0.5– 1.5 mV) **(Table 1)**.^28^ Once the low-voltage “scar” areas have been identified, pace mapping along the scar border zone (0.5–1.5 mV) can then be performed to approximate the “exit” site of the VT circuit. At the putative “exit,” the paced QRS morphology should be similar to the spontaneous arrhythmia, with a short stimulus-QRS interval (≤ 40 ms). Once identified, further mapping efforts can be made into the scar region and away from the border zone, where a critical isthmus is often located.

### Electrically unexcitable scar

Electrically unexcitable scar (EUS) is defined by areas of high-output unipolar pacing (< 10 mA at 2-ms pulse width) in regions of low amplitude electrograms (< 0.5 mV) that fail to capture myocardium. EUS has been correlated with areas of dense scar with local bipolar voltages of < 0.25 mV. Since areas of EUS are commonly located in proximity to VT isthmuses and potential conduction channels, a potential VT isthmus is often located in between areas of EUS.^29^

### Conducting channels

The histology of an endocardial resection specimen from post-infarction patients with sustained VT revealed isolated bundles of viable myocardial fibers within the infarct tissue, often associated with fractionation of electrograms and slow conduction.^8,9^ These potential conduction channels can be identified in the majority of patients by making careful adjustments to the voltage color gradient of the bipolar voltage maps of lowvoltage areas (< 1.5 mV) during baseline sinus or paced rhythm. A “conducting channel” is defined by the presence of a “corridor” of consecutive electrograms differentiated by higher bipolar voltage amplitudes than the surrounding tissue.^28,30^ However, a single cutoff threshold value is often not sufficient due to the heterogeneous nature of the scar tissue, and frequent adjustments of voltage color thresholds are often required to visualize potential channels.

**Figure 3:**
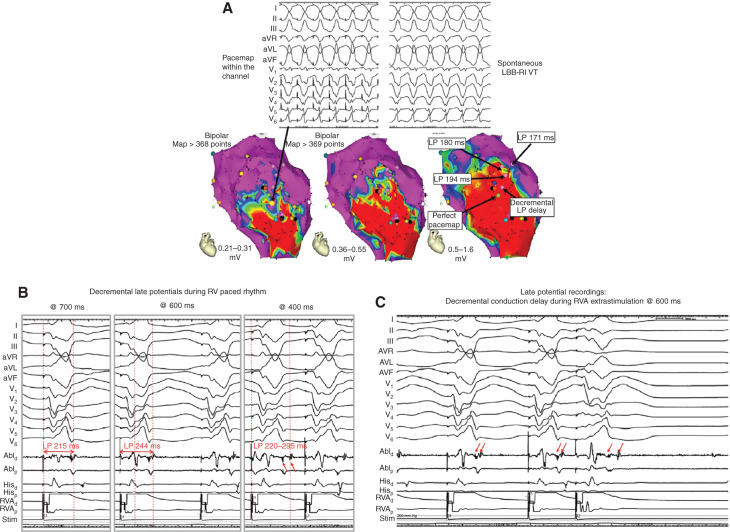
Identification of VT-conducting channel. **A:** Catheter mapping in a patient with a large anterior myocardial infarction. The spontaneous VT has a left bundle branch block–right inferior (LBB-RI) QRS morphology. The endocardial bipolar voltage map showed a large anterior scar. The color gradient adjustments of the bipolar voltage correspond with 0.5 mV to 1.6 mV, 0.35 mV to 0.55 mV, and 0.21 mV to 0.31 mV. A potential VT-related conduction channel can be identified at a color threshold range of 0.21 mV to 0.31 mV. Recordings from inside this channel during differential pacing demonstrate progressively late potentials from the border zone into the scar area. Pacing from within this channel with decremental late potentials/LAVAs resulted in a perfect pacemap suggestive of a potential VT isthmus site. **B:** With right ventricular pacing at different rates, delayed and fractionated signals were recorded within the channel. Decremental prolongation of the pacing stimuli-to-potential intervals were noted with increasing pacing rates, suggestive of local conduction delay into the channel. **C:** With right ventricular premature stimulation, decremental multicomponent and fractionated local electrograms were also noted within the channel, suggesting slow conduction into the potential VT isthmus site. LAVAs: local abnormal ventricular activities; LP: late potential; VT: ventricular tachycardia. Adapted from Al-Ahmad AA, Callans DJ, Hsia HH, et al. *Hands-On Ablation: The Experts’ Approach*. 2^nd^ ed. Minneapolis, MN: Cardiotext Publishing, LLC; 2017. Used with permission from Cardiotext Publishing.

A majority of VT-related conducting channels can be identified in areas of scar when the bipolar voltage threshold is set to between 0.2 mV and 0.3 mV **(Figure 3)**.^30^ These channels have been correlated with the critical VT isthmuses by entrainment mapping,^28^ and can be identified non-invasively by contrast-enhanced MRI.^31^ The presence of late or fractionated potentials within the channel significantly improves its specificity for identifying VTrelated isthmus.^32^ Further mapping efforts are needed to establish the functional significance of these VT-related channels, such as activation mapping, pace mapping, and entrainment mapping.

### Late potentials and local abnormal ventricular activity

LAVA is a general term that incorporates all abnormal ventricular electrograms that represent near-field signals recorded from non-uniform anisotropic slow conduction of surviving viable myocardial fibers within the potential VT isthmus.^33,34^ Most LAVAs reside in scar or border zones, and may appear as late potentials or electrograms with isolated delayed components recorded after the ventricular activation/surface QRS.^35,36^

In patients with post-infarction VTs, the endocardial low voltage scar (< 1.5 mV) and the distribution of LAVA recordings have been located in regions of myocardial wall thinning (< 5 mm) on contrast-enhanced computed tomography (CT). Very late LAVA (> 100 ms after QRS complex) were almost exclusively (93%) detected within the thinnest scar area (< 3 mm thickness).^37^ Integration of contrast-enhanced CT imaging with three-dimensional electroanatomic mapping helps to guide substrate-based ablation in post-infarction VTs.

However, the latency and the ability to detect LAVA/late potentials are dependent on the activation wavefronts with variable fusion of near-field versus far-field signals, and thus are affected to a large extent by the anatomical locations of scar. LAVAs were more frequently detected after the QRS complex in the epicardium (91%) than in the endocardium (66%; p < 0.001), and only 43% of septal LAVA were detectable and separated from far-field ventricular electrograms.^38^ Ventricular pacing or pacing at different rates provides different wavefront propagations that may unmask areas of slow conduction/LAVA **(Figure 3B)**.^30,39^ Delivery of ventricular extrastimuli also can reveal decremental impulse propagation in areas of slow conduction demonstrating increased fractionation of LAVA/late potential signals **(Figure 3C)**. In patients with left ventricular (LV) cardiomyopathies/VT substrates, LV stimulation may be more useful than right ventricular (RV) pacing in unmasking LAVA. This may be achieved by either inserting a catheter into a LV venous branch via the coronary sinus or by having simultaneous retrograde and trans-septal LV access for LV stimulation and recording.

### Pacemapping

**Figure 4:**
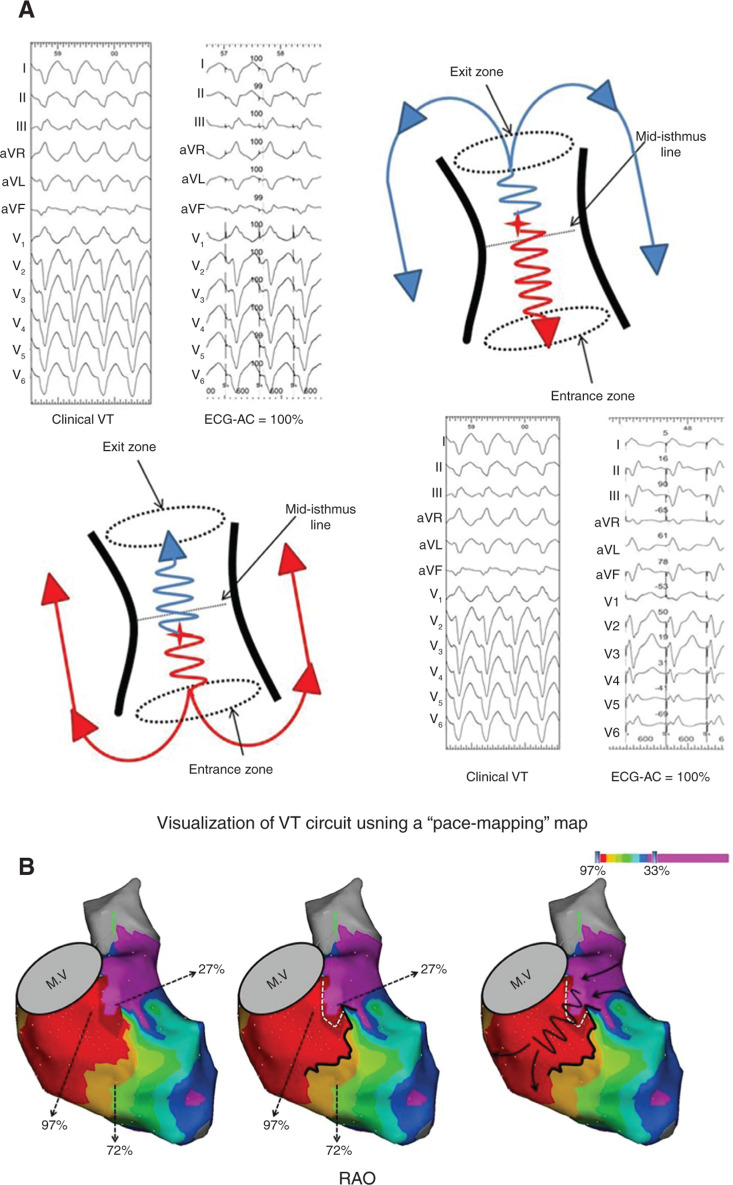
Pacemap localization of VT circuit. **A:** A schema of a “figure-of-eight” reentrant VT circuit. During sinus rhythm, when pacing at the exit side of the mid-isthmus line, the activation wavefront propagates in two directions, but more rapidly to the exit site, resulting in near-identical RS morphologies as that of clinical VTs with an ECG average correction (ECG-AC) of 100%. When pacing at the entrance side of the mid-isthmus, the wavefront propagates more rapidly to the entrance and away from the exit, resulting in different ventricular activation patterns and paced QRS morphologies, with a poor ECG correlation (ECGAG: 10%). **B:** Left: An example of a VT circuit visualized using a “pacemapping” map superimposed on the three-dimensional electroanatomic maps. The color gradient corresponds with the degree of ECG correlation as compared with the clinical VT (> 97% pacemap QRS match: red; < 33% QRS match: purple). Middle: A pacemapping map showing a good (97%) correlation zone, corresponding with the VT exit. Close to this zone, a poor (27%) correlation zone with an abrupt transition within a short distance is seen. In another direction, an abrupt transition is observed between the good correlation zone and a zone with an intermediate correlation (92%). Right: the abrupt transition line between the very good and the bad correlation zones defines the mid-isthmus line (white dashed line), while transition between the very good and intermediate correlation zones defines the lateral boundary of the isthmus (black line). ECG: electrocardiogram; MV: mitral valve; RAO: right anterior oblique; VT: ventricular tachycardia. Adapted from Al-Ahmad AA, Callans DJ, Hsia HH, et al. *Hands-On Ablation: The Experts’ Approach*. 2^nd^ ed. Minneapolis, MN: Cardiotext Publishing, LLC; 2017. Used with permission from Cardiotext Publishing.

Pacemapping is also useful in localizing the critical isthmus of post-infarction VTs.^40^ Topographically, when pacing at the exit side of the isthmus of the VT circuit, the activation wavefront propagates more rapidly to the exit site, resulting in a good ECG match as that of the clinical VT. In contrast, when pacing at the entrance side of the VT isthmus, the activation propagates preferentially out of the entrance site, resulting in a very different paced QRS morphology and a poor QRS match compared with the clinical VT **(Figure 4A)**.

High-density pacemapping (bipolar or unipolar) is performed within abnormal low-voltage regions during sinus rhythm. Conceptually, stepwise pacemapping from the exit to the isthmus should lead to a gradual prolongation of the S-QRS interval while maintaining a nearperfect QRS matching to the VT. Importantly, an abrupt transition from an area of perfect pacemapping to an area of poor matching (such as the entrance side of the isthmus) corresponds with the VT isthmus. By quantifying the degree of paced QRS match at each location using a color gradient, this identifies the virtual transition zone (from the best to the worst matching sites), and thus the course of the VT circuit can be characterized **(Figure 4B)**.

High-density multielectrode mapping VT substrate characterization has evolved with the emergence of highdensity multielectrode mapping catheters, including the PentaRay^®^ (Biosense Webster, Diamond Bar, CA, USA); the Relexion™ HD spiral catheter and the DuoDeca Livewire™ catheter (Abbott Laboratories, Chicago, IL, USA); and the IntellaMap Orion™ catheter and/or Rhythmia™ mapping system (Boston Scientific, Natick, MA, USA).^41–44^ The local electrogram voltages and morphologies are dependent on the recording electrode size, interelectrode spacing, and direction of wavefront propagation.^44,45^ When compared with the standard ablation catheter with the 3.5 mm to 4.0 mm distal electrode, tissue characterization using a catheter with small (1.0 mm), closely spaced electrodes (PentaRay^®^; Biosense Webster, Diamond Bar, CA, USA) resulted in a 22% smaller low-voltage area (< 1.5 mV) and a 47% reduction in dense scar size (< 0.5 mV) in an animal model of infarction.^42^ High-density, multielectrode mapping minimizes the effects of farfield signals and helps to identify heterogeneity within the low-voltage scar, allowing for better localization of channels of surviving myocardial bundles and fractionated electrograms or LAVA detected within these channels.^41,46^

The use of multipolar catheter mapping is associated with a higher mapping density with better endocardial LAVA identification in patients with post–myocardial infarction VT. Overall, the impact of multipolar catheter use on patient outcomes seems to be mediated by a more comprehensive characterization of the VT substrate, which translated to a lower VT recurrence rate following catheter ablation.^41,47^

## Ischemic cardiomyopathy versus nonischemic cardiomyopathy

In patients with post-infarction VT, endocardial scar extension and density predict scar transmurality and the endoepicardial presence of LAVA. The presence of LAVA predicts a favorable VT ablation outcome. However, endocardial bipolar voltage may underestimate the scar size as compared with MRI, possibly due to the presence of hibernating myocardium, early reperfusion, collateral circulation, or a concomitant cardiomyopathy.^48^ Importantly, the size of the endocardial unipolar lowvoltage area surrounding the bipolar scar (penumbra) reflects the intramural/epicardial scar and diffuse myocyte loss. This is associated with an increased mortality and arrhythmia recurrence following ablation.^48,49^

**Figure 5:**
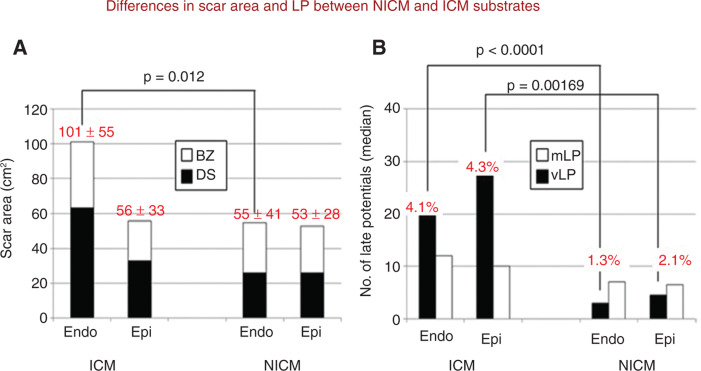
Differences in scar area and late potentials between ICM and NICM substrates. **A:** With high-density electroanatomical maps of patients with ICM, the dense scar was twice as large on the endocardium as compared with on the epicardium. Such predilection for endocardial scar was not as prominent in patients with NICM, with nearly equal extents of scar on the endocardium and epicardium present. Less dense scar (solid bars) was observed in patients with NICM. The open bars indicate the border zone. **B:** Patients with ICM had more late potentials and evidence of slow conduction than patients with NICM. This difference was driven by a greater number of very late potentials (vLPs) (solid bars) in ICM. BZ: border zone; DS: dense scar; ICM: ischemic cardiomyopathy; mLP: moderate late potentials; NICM: nonischemic cardiomyopathy. Modified from Nakahara S, Tung R, Ramirez RJ, et al. Characterization of the arrhythmogenic substrate in ischemic and non-ischemic cardiomyopathy. *J Am Coll Cardiol.* 2010;55(21):2355–2365. Used with permission.

In comparison with post-infarction VT, patients with NICM have no predilection for endocardial scar substrate, with a nearly equal extent of scar occurring on the endocardium and epicardium, respectively. Importantly, less dense scar (< 0.5 mV) was observed in patients with NICM, and late or fractionated potentials refl ecting conduction delay are significantly less prevalent **(Figure 5)**. In these cases, a late potential/LAVA-guided ablation strategy is associated with less favorable outcomes than post-infarction patients.^50^

The majority of NICM patients who failed prior endocardial ablations often have more extensive areas of abnormal voltage on the epicardium rather than on the endocardium, and an epicardial ablation may be needed in up to 50% of these individuals.^20,51^ However, the distribution and extent of endocardial versus epicardial scars may vary in individual patients, and the utility of endocardial voltage mapping in identifying non- endocardial scar is limited.^52^ Unipolar recordings provide a larger field of view than bipolar recordings, allowing for the detection of epicardial or intramural scars during endocardial mapping. Based on clinical and MRI data, a cutoff value of unipolar endocardial voltage of ≤ 6.78 mV and < 8.27 mV best differentiated the presence of underlying intramural and epicardial scars, respectively.^53,54^

**Figure 6:**
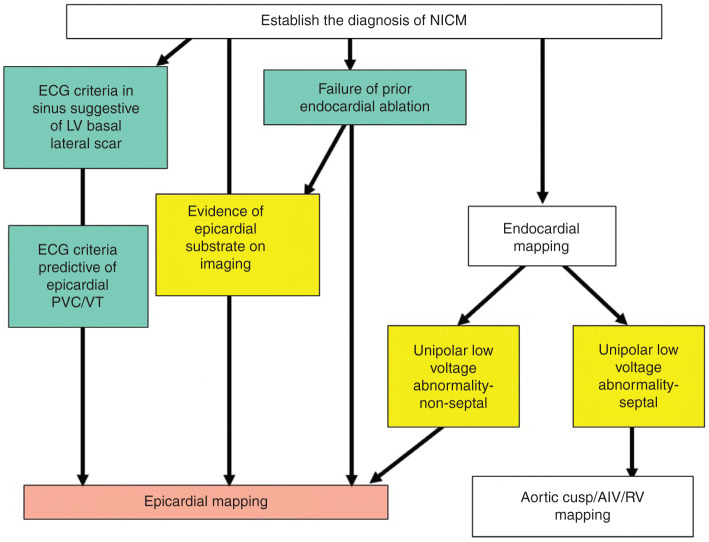
A VT ablation approach in patients with NICM. This figure represents a flow chart and a potential approach in patients with NICM who undergo catheter ablation for VT. The ECG criteria during sinus rhythm that predict the presence of LV basal lateral scar include an R-wave in lead V1 ≥ 0.15 mV and an S-wave in lead V6 ≥ 0.15 mV.^76^ This can be distinguished from patients with prior inferior/inferolateral myocardial infection by (1) lateral lead QRS fragmentation, (2) a lack of inferior Q waves, and (3) a lead V6 S/R ratio of ≥ 0.25.^77^ These findings may be useful in predicting the presence of LV scar and in determining the risk of VT in patients with NICM. The ECG criteria predictive of an epicardial exit during ventricular arrhythmias (ie, PVCs, VTs) are summarized in **Table 2**. Epicardial mapping may be considered in selected patients when (1) the 12-lead ECG of the VT suggests an epicardial origin; (2) there is evidence of epicardial substrate on imaging studies (eg, MRI, intracardiac echocardiography); (3) there is unipolar electrogram voltage abnormality; and (4) there is failure of prior endocardial ablation. The important decision steps are highlighted in yellow. AIV: anterior interventricular vein; ECG: electrocardiogram; LV: left ventricle; MRI: magnetic resonance imaging; NICM: nonischemic cardiomyopathy; PVC: premature ventricular complexes; RV: right ventricle; VT: ventricular tachycardia.

Recent studies of electroanatomical mapping and MRI use in patients with NICM demonstrated two predominant scar patterns, specifically (1) basal anteroseptal and (2) inferolateral locations of nearly equal distribuions.^55,56^ In the subset of patients with inferolateral scars, epicardial VT substrate with late potentials were frequently (63%–81%) identified. In contrast, the arrhythmia substrate in patients with the anteroseptal scar subtype were mainly identified in the periaortic and intramural septal locations, and were associated with more extensive endocardial unipolar scar and a higher VT recurrence rate. These .ndings provide a better understanding of the arrhythmogenic substrate in patients with NICM and the prognostic significance. Epicardial mapping may be considered in selected patients when (1) the 12-lead ECG of the VT suggests an epicardial origin **(Table 2)**; (2) there is evidence of epicardial substrate on imaging studies (eg, magnetic resonance, intracardiac echocardiography); (3) unipolar electrogram voltage abnormality in the presence of no or minimal bipolar (< 1.5 mV) endocardial scar; and (4) failure of prior endocardial ablation **(Figure 6)**.

## Ablation strategies

The primary goal of catheter ablation of scar-related VT is the interruption of critical areas of slow conduction responsible for the development and maintenance of the reentrant circuit. Most patients with scar-related VT present with unstable arrhythmias that are not amenable to conventional mapping techniques based on point-by-point activation mapping or overdrive entrainment. In these scenarios, a “substrate-based” ablation strategy facilitates workflow, optimizes lesion sets, and improves patient safety and procedural outcomes.

**Figure 7:**
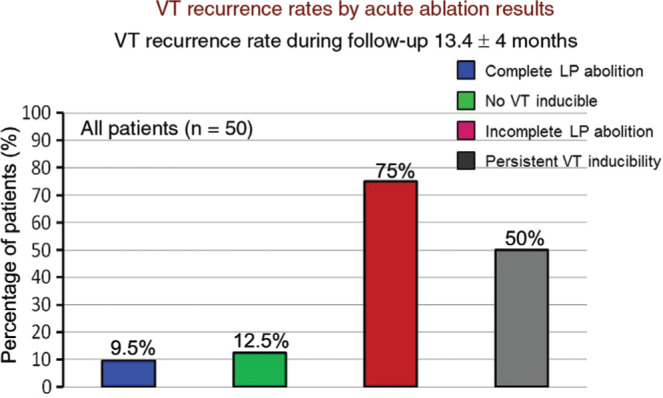
VT recurrence rate by acute ablation results. In patients with post-infarction VT, successful late potential abolition and post-procedural VT non-inducibility constitute significant endpoints after catheter ablation. Complete late potential abolition or elimination reduces VT recurrence to exceptionally low rates and compares favorably with post-procedural non-inducibility of VT. Persistence of late potentials/LAVAs or persistent inducibility predict a poor outcome. LAVAs: local abnormal ventricular activities; LP: late potential; VT: ventricular tachycardia.

Various substrate modification techniques have been described for unmappable or hemodynamically intolerable VT. Linear radiofrequency lesions are usually created to transect the potential isthmuses or channels, or to connect the VT exit sites to anatomic barriers such as the EUS or a valvular annulus. An ablation strategy guided by abnormal potentials, coupled with local voltage profile analysis **(Table 1)**, has been shown to be an effective strategy of VT control in patients with structural heart disease, particularly in those with ICM.^47,48,57^ With high-density mapping and extensive ablations across the endocardial ± epicardial surfaces, the elimination of all LAVA may be achieved in ~70% of patients.^57,58^ Complete LAVA elimination is associated with a reduction in recurrent VT or death (p = 0.035) compared with the persistence of LAVA **(Figure 7)**, and represents a useful and effective procedural endpoint for substrate-based VT ablation. Although non-inducibility predicts a lower risk of VT occurrence, a combined endpoint of LAVA elimination of VT non-inducibility is associated with the lowest VT recurrence rate (16%), in comparison with non-inducibility alone.^58^

However, the success of such potential guided ablation strategies may depend on the substrate location and the patient’s anatomy.^38,59^ In some individuals, LAVA could not be identifi ed or eliminated due to intramural or epicedial scar; septal (or early activated) substrate locations; inadequate sampling; or difficult anatomical characteristics, such as the papillary muscles.

**Figure 8:**
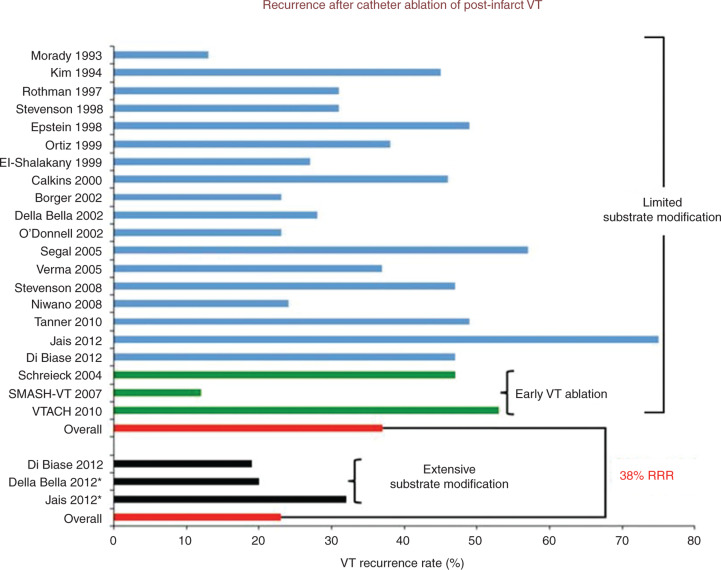
Recurrence of post-infarction VT after catheter ablation. The blue bars represent studies that used a limited substrate ablation approach. The green bars represent studies that evaluated early intervention with a limited substrate ablation. The black bars represent studies utilizing an extensive substrate-based modification ablation approach. The pooled data are depicted as red bars. *Studies that included a subgroup of patients with NICM. RRR: relative risk reduction; VT: ventricular tachycardia. Adapted from Al-Ahmad AA, Callans DJ, Hsia HH, et al. *Hands-On Ablation: The Experts’ Approach.* 2^nd^ ed. Minneapolis, MN: Cardiotext Publishing, LLC; 2017. Used with permission from Cardiotext Publishing.

The presence of a complex arrhythmia substrate with multiple potential conducting channels and possible midwall or epicardial VT exit sites provided the rationale for an extensive endocardial (and perhaps epicardial) ablation strategy in those with structural heart disease. Such “scar homogenization” increases the freedom from arrhythmia at long-term follow-up in patients with post-infarction VT storm.^60^ Other novel ablation strategies include “core isolation” and “scar dechanneling.” Core isolation involves identifying an area of interest within dense scar that includes areas of voltage channels, late potentials, and isthmus sites. Contiguous ablation lesions are created to either completely surround a particular region or to anchor it to nearby anatomic structures, with the endpoint of exit block or inexcitability within this core area.^61^ Scar dechanneling represents an alternative method to LAVA abolition with focal ablation performed at sites at which the earliest fractionated potentials are recorded near the entrance of the conducting channels, aiming toward the endpoint of eliminating a consecutive series of LAVA.^62^ These substrate-based ablation approaches may be particularly useful in patients who are non-inducible at baseline or in those who have multiple, unmappable arrhythmias. Importantly, these techniques are achievable in most patients and represent definable procedural endpoints. Overall, an extensive substrate-based ablation strategy is associated with a 38% relative risk reduction in VT recurrence as compared with limited substrate modification **(Figure 8)**.^63^

Real-time image integration to guide catheter ablation is feasible in large series of patients with scar-related VTs, facilitating workflow with significant impacts on procedural management with improved outcomes. Imaging integration provides important complementary information to electroanatomical mapping, particularly in patients with NICM or in those undergoing epicardial ablations.^47,64^ The structural abnormality may be segmented as areas of delayed gadolinium enhancement on MRI, and/or as areas of wall thinning on multidetector CT scan, and are correlated with local conduction delay defined by late potentials or LAVA.^37,65^ Real-time image integration for substrate localization/characterization for VT ablation will soon become an accepted standard.

## Outcomes

The impact of technical innovations and substrate-based ablation approaches on VT ablation has recently been evaluated.^47^ Complete LAVA elimination, the use of real-time image integration, and high-density mapping with multipolar catheters were independent predictors of VT free survival after catheter ablation for post-myocardial infarction VT **(Figure 7)**. These findings strongly suggest that complete LAVA elimination is a predictive procedural endpoint associated with a twofold reduction in VT recurrence.

However, catheter ablation of ventricular arrhythmias in the setting of non-ischemic dilated cardiomyopathy remains a major challenge **(Table 3)**. A substrate-based ablation approach is less applicable, and a greater proportion of patients require epicardial ablation. The ablation procedures are associated with significantly longer operation time and fluoroscopic exposure, with a lower long-term cumulative VT-free survival than post-infarction VTs.^66^ Serial electroanatomic mapping also demonstrated new voltage abnormalities in approximately 50% of NICM patients with VT, often accompanied by progressive ventricular remodeling.^67,68^ Despite these limitations, an improvement in overall VT burden with a reduced need for antiarrhythmic drugs can be achieved with aggressive endocardial and adjuvant epicardial ablation. Catheter ablation remains a safe and effective approach for longterm arrhythmia control in most NICM patients.^69^

The International VT Ablation Center Collaborative Group represents the largest retrospective multicenter study that demonstrated that freedom from VT recurrence after catheter ablation was associated with an improved transplant-free survival, independent of ejection fraction, heart failure, and type of cardiomyopathy (ICM: 89% versus 72%; p < 0.001 and NICM: 92% versus 72%; p < 0.001) in patients with structural heart disease.^70^ The recently published Ventricular Tachycardia Ablation versus Escalated Antiarrhythmic Drug Therapy in Ischemic Heart Disease (VANISH) trial prospectively compared the use of antiarrhythmic drugs containing amiodarone and/ or mexiletine with catheter ablation. A significantly lower rate of composite primary outcomes of death, VT storm, or appropriate ICD shock were observed in patients who underwent catheter ablation versus those who received an escalating dosage of amiodarone or the addition of mexiletine.^71^ However, no significant difference was observed among patients who were not treated with amiodarone at baseline. Nonetheless, these findings support the preemptive use of early catheter ablation in the management of ventricular arrhythmias in patients with structural heart disease refractory to antiarrhythmic drugs.

## Utilities of hemodynamic support

However, catheter ablation of VT in patients with NICM remains a challenge compared with that of post-infarction patients. Imaging integration coupled with a systemic, detailed substrate characterization has shown promise in providing a safe and effective approach for long-term arrhythmia control in most NICM patients. Despite a shift towards a more substrate-based ablation strategy, activation and entrainment mapping helps to identify the critical isthmus and to avoid potential unnecessary ablations. However, hemodynamic instability limits such conventional mapping techniques in the majority of patients, and is associated with an increased mortality.^72–74^

Percutaneous left ventricular assist devices (pLVADs) commonly used in VT ablation include the TandemHeart™ left atrial-to-femoral artery bypass device (CardiacAssist Inc., Pittsburgh, PA, USA) and the Impellas left ventricle-toascending aorta pump (Abiomed, Danvers, MA, USA). The use of pLVADs during unstable VT in patients with structural heart disease appears to be feasible and safe. pLVADs provide adequate end-organ perfusion during VTs, facilitate extensive mapping, and require fewer rescue shocks during the procedure. However, improvement in long-term procedural outcome has not been demonstrated. The use of hemodynamic support during VT ablation therefore needs to be weighed against the risk of complications, longer procedure time, and the incremental cost.^73–75^

## Summary

Ventricular arrhythmias arise from complex electroanatomical substrates and are a significant cause of morbidity and mortality in patients with structural heart disease. Catheter ablation is historically implemented as a treatment of last-resort. There have been recent advancements in mapping technologies and techniques allowing for better definition of potential surrogate targets of the VT substrate. Various substrate-based mapping and ablation strategies such as targeting LAVA, along with homogenization or dechanneling strategies improve the procedural outcomes and minimize complications. A systematic approach to mapping and ablation of VT is paramount, and catheter ablation has shifted to a potential first-line therapy for early intervention purposes, particularly in patients with post-infarction VTs.

## Figures and Tables

**Table 1:** Local Electrogram Amplitude for Sites within the Reentrant Circuit.

	Entrance	Central Isthmus	Exit	Outer Loop
Dense scar (< 0.5 mV)	17	30	18	6
Border zone (0.5–1.5 mV)	2	7	26	18
Normal (> 1.5 mV)	–	–	4	8
Total (136 sites)	19	37	48	32

Modified with permission from: Hsia HH, Lin D, Sauer WH, Callans DJ, Marchlinski FE. Anatomic characterization of endocardial substrate for hemodynamically stable reentrant ventricular tachycardia: identification of endocardial conducting channels. *Heart Rhythm.* 2006;3(5):503–512.

**Table 2:** ECG Criteria Predictive of An Epicardial Exit of Ventricular Arrhythmias

Author (Year)	Ventricular Region	ECG Criteria Predictive of Epicardial Exit	Sensitivity	Specificity
Berruezo et al.^22^	LV	Precordial Pseudo-delta wave = 34 ms	83%	95%
		Intrinsicoid deflection time in V2 = 85 ms	87%	90%
		Shortest precordial RS duration = 121 ms	76%	85%
Bazan et al.^78^	Inferior RV	Q waves in inferior leads	71%	74%
	Anterior exit	Q wave in lead I	52%	94%
		QS complex in V2	67%	61%
Bazan et al.^79^	Basal-superior LV	Q wave in lead I	86%	81%
	Apical-superior LV	Q wave in lead I	84%	74%
	Basal-inferior LV	Q waves in inferior leads	74%	51%
	Apical-inferior LV	Q waves in inferior leads	94%	61%
Valles et al.^80^	Basal superior and lateral LV	Algorithm with the parameters of: 1. absence of Q waves in inferior leads; 2. precordial pseudo-delta wave = 75 ms; 3. precordial MDI = 0.59; and 4. q wave in lead I.	Pacemap: 96% VT: 88%	Pacemap: 93% VT: 88%

ECG: electrocardiogram; MDI: maximum deflection index; RV: right ventricle; LV: left ventricle; VT: ventricular tachycardia.

**Table 3:** Heart Centre of Leipzig VT (HELP-VT) Study Results for VT Ablation in NICM versus ICM

	NICM (n = 63)	ICM (n = 164)	p-Value
Epicardial ablation, n (%)	19 (30.2%)	2 (1.2%)	0.0001
Noninducible PES, n (%)	9 (15.8%)	14 (9.9%)	0.36
Substrate mapping, n (%)	42 (66.7%)	147 (89.6%)	< 0.001
VT induced, n/pt	2.1 ± 1.2	2.2 ± 1.3	0.74
VT mappable, n/pt	1.6 ± 0.8	2.0 ± 0.8	0.06
VT ablated, n/pt	1.4 ± 1.1	1.6 ± 1.2	0.17
Clinical VT cycle length (ms)	364 ± 86	385 ± 93	0.133
Procedure time (min)	181 ± 64	155 ± 49	0.003
Fluoroscopy time (min)	39 ± 22	26 ± 19	0.0001
Failure, n (%)	7 (11.1%)	8 (4.9%)	0.13

VT: ventricular tachycardia; n/pt: number of patients; NICM: non-ischemic cardiomyopathy, ICM: ischemic cardiomyopathy; PES: programmed electric stimulation. Adapted from Dinov B, Fiedler L, Schönbauer R, et al. Outcomes in catheter ablation of ventricular tachycardia in dilated nonischemic cardiomyopathy compared with ischemic cardiomyopathy: results from the Prospective Heart Centre of Leipzig VT (HELP-VT) study. *Circulation*. 2014;129(7):728–736.
